# Deciphering OPDA Signaling Components in the Momilactone-Producing Moss *Calohypnum plumiforme*

**DOI:** 10.3389/fpls.2021.688565

**Published:** 2021-05-31

**Authors:** Hideo Inagaki, Koji Miyamoto, Noriko Ando, Kohei Murakami, Koki Sugisawa, Shion Morita, Emi Yumoto, Miyu Teruya, Kenichi Uchida, Nobuki Kato, Takuya Kaji, Yousuke Takaoka, Yuko Hojo, Tomonori Shinya, Ivan Galis, Akira Nozawa, Tatsuya Sawasaki, Hideaki Nojiri, Minoru Ueda, Kazunori Okada

**Affiliations:** ^1^Graduate School of Science and Engineering, Teikyo University, Utsunomiya, Japan; ^2^Department of Biosciences, Faculty of Science and Engineering, Teikyo University, Utsunomiya, Japan; ^3^Advanced Instrumental Analysis Center, Teikyo University, Utsunomiya, Japan; ^4^Agro-Biotechnology Research Center, Graduate School of Agricultural and Life Sciences, The University of Tokyo, Tokyo, Japan; ^5^Graduate School of Science, Tohoku University, Sendai, Japan; ^6^Institute of Plant Science and Resources, Okayama University, Kurashiki, Japan; ^7^Proteo-Science Center, Ehime University, Matsuyama, Japan; ^8^Collaborative Research Institute for Innovative Microbiology, The University of Tokyo, Tokyo, Japan; ^9^Graduate School of Life Sciences, Tohoku University, Sendai, Japan

**Keywords:** oxylipin, plant hormone, 12-oxo-phytodienoic acid, moss, *Calohypnum plumiforme*

## Abstract

Jasmonic acid (JA) and its biologically active form jasmonoyl-L-isoleucine (JA-Ile) regulate defense responses to various environmental stresses and developmental processes in plants. JA and JA-Ile are synthesized from α-linolenic acids derived from membrane lipids *via* 12-oxo-phytodienoic acid (OPDA). In the presence of JA-Ile, the COI1 receptor physically interacts with JAZ repressors, leading to their degradation, resulting in the transcription of JA-responsive genes by MYC transcription factors. Although the biosynthesis of JA-Ile is conserved in vascular plants, it is not recognized by COI1 in bryophytes and is not biologically active. In the liverwort *Marchantia polymorpha*, dinor-OPDA (dn-OPDA), a homolog of OPDA with two fewer carbons, and its isomer dn-*iso*-OPDA accumulate after wounding and are recognized by COI1 to activate downstream signaling. The moss *Calohypnum plumiforme* produces the antimicrobial-specialized metabolites, momilactones. It has been reported that JA and JA-Ile are not detected in *C. plumiforme* and that OPDA, but not JA, can induce momilactone accumulation and the expression of these biosynthetic genes, suggesting that OPDA or its derivative is a biologically active molecule in *C. plumiforme* that induces chemical defense. In the present study, we investigated the biological functions of OPDA and its derivatives in *C. plumiforme*. Searching for the components potentially involving oxylipin signaling from transcriptomic and genomic data revealed that two *COI1*, three *JAZ*, and two *MYC* genes were present. Quantification analyses revealed that OPDA and its isomer *iso*-OPDA accumulated in larger amounts than dn-OPDA and dn-*iso*-OPDA after wounding. Moreover, exogenously applied OPDA, dn-OPDA, or dn-*iso*-OPDA induced the transcription of *JAZ* genes. These results imply that OPDA, dn-OPDA, and/or their isomers potentially act as biologically active molecules to induce the signaling downstream of COI1-JAZ. Furthermore, co-immunoprecipitation analysis showed the physical interaction between JAZs and MYCs, indicating the functional conservation of JAZs in *C. plumiforme* with other plants. These results suggest that COI1-JAZ-MYC mediated signaling is conserved and functional in *C. plumiforme*.

## Scheme 1

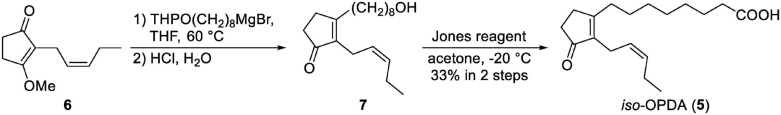

## Introduction

The phytohormone jasmonic acid (JA; **1**) and its biologically active form jasmonoyl-L-isoleucine (JA-Ile) regulate defense responses to various environmental stresses such as wounding, and plant growth and development (Wasternack and Hause, 2013; Wasternack, 2015; Guo et al., 2018; Ballaré and Austin, 2019). JA and JA-Ile are synthesized from fatty acids, α-linolenic acid or hexadecatrienoic acid, derived from membrane lipids. Oxygenation by lipoxygenase and dehydration-cyclization by allene oxide synthase and allene oxide cyclase (AOC) converts α-linolenic acid to 12-oxo-phytodienoic acid (OPDA; **2**). The double bond hydrogenation by OPDA reductase (OPR3) and three sequential β-oxidations convert OPDA to JA (Wasternack and Hause, 2013). Finally, the GH3 family protein JAR1 conjugates JA to isoleucine to produce the biologically active form (+)-7-*iso*-JA-Ile (Staswick and Tiryaki, 2004; Fonseca et al., 2009).

JA-Ile is recognized by the F-box protein COI1 (Xie et al., 1998; Yan et al., 2009; Sheard et al., 2010). JAZ proteins repress transcription factors such as MYC, which activate the transcription of JA-responsive genes, when JA-Ile is at low levels. In the presence of high JA-Ile levels, COI1 physically interacts with JAZ repressors, leading to their degradation, and the transcription of JA-responsive genes by MYC and other transcription factors (Chini et al., 2007; Thines et al., 2007).

Although its biosynthesis is conserved in vascular plants (Wasternack and Hause, 2013; Wasternack, 2015; Pratiwi et al., 2017), JA-Ile is not a biologically active form recognized by COI1 in bryophytes. In the liverwort *Marchantia polymorpha*, 2,3-dinor-OPDA (dn-OPDA; **3**) and its isomer 2,3-dinor-12-oxo-9(13),15(*Z*)-phytodienoic acid (dn-*iso*-OPDA; **4**) accumulate after wounding, whereas JA-Ile is not found. dn-OPDA and dn-*iso*-OPDA are recognized by MpCOI1 to activate downstream signaling (Monte et al., 2018). The model moss *Physcomitrium patens*, formerly known as *Physcomitrella patens*, also produces dn-OPDA and dn-*iso*-OPDA (Stumpe et al., 2010; Mukhtarova et al., 2020). Additionally, 12-oxo-9(13),15(*Z*)-phytodienoic acid (*iso*-OPDA; **5**) has been found in *P. patens*, but its biological activity has not been revealed (Mukhtarova et al., 2020). Comparative genomics and metabolomics suggest that dn-OPDA and dn-*iso*-OPDA might act as COI1 ligands in mosses (Monte et al., 2018; Mukhtarova et al., 2020).

The moss *Calohypnum plumiforme* like rice and barnyard grass produces specialized metabolites, momilactones, which have antimicrobial and allelochemical activities (Nozaki et al., 2007; Yamane, 2013; Miyamoto et al., 2014; Guo et al., 2017). These momilactone-producing plants possess the momilactone biosynthetic gene cluster, which is formed by the concentration of biosynthetic genes in a narrow region of the chromosome (Shimura et al., 2007; Guo et al., 2017; Mao et al., 2020). Therefore, the investigation of the regulatory mechanism(s) of momilactone production in *C. plumiforme* will contribute to a better understanding of the diversity and evolution of plant chemical defenses. Previous studies have revealed that JA and JA-Ile are not detected in *C. plumiforme* and that OPDA, but not JA, induces momilactone accumulation and the expression of these biosynthetic genes (Okada et al., 2016; Mao et al., 2020). These results suggest that OPDA or its derivative is a biologically active molecule in *C. plumiforme* that induces the production of momilactones.

In the present study, we focused on the function of OPDA and its derivatives and explored the potential involvement of protein components in oxylipin signaling in the moss *C. plumiforme*. We found two *COI1*, three *JAZ*, and two *MYC* genes from the transcriptome and genome data. The quantification of endogenous oxylipins revealed that OPDA and *iso*-OPDA accumulated in larger amounts than dn-OPDA and dn-*iso*-OPDA. We also found that exogenously applied OPDA, dn-OPDA, or dn-*iso*-OPDA induced the transcription of *JAZ* genes. Moreover, co-immunoprecipitation analysis revealed the physical interaction between JAZs and MYCs. These results imply that OPDA, dn-OPDA, and/or their isomers act as biologically active molecules to induce downstream signaling and that COI1-JAZ-MYC-mediated signaling is conserved in *C. plumiforme*.

## Materials and Methods

### Plant Materials and Growth Conditions

*Calohypnum plumiforme* kindly provided by Prof. Kenichiro Hayashi (Okayama University of Science) was the same line as that used in a previous report (Okada et al., 2016). Gametophores were cultured on BCDATG agar medium under continuous white light at 23°C (Nishiyama et al., 2000), transferred to a new BCDATG agar medium every 2 months, and used for chemical and wounding treatments 1 month after each transfer.

### Chemicals

Racemic JA was purchased from Tokyo Chemical Industry Co. (Tokyo, Japan) and used as a standard for quantification analysis. (–)-JA was prepared as previously described (Miyamoto et al., 2019) and used for the treatment of *C. plumiforme*. [^2^H_5_]-JA was obtained from Nacalai Tesque, Inc. (Kyoto, Japan). (–)-JA-Ile and [^13^C_6_]-JA-Ile were synthesized as previously described (Jikumaru et al., 2004).

OPDA and [^2^H_5_]-OPDA were purchased from Olchemim (Olomouc, Czechia Republic) and used as standards for quantification analysis. For the treatment of *C. plumiforme*, OPDA and [^2^H_5_]-OPDA were synthesized from α-linolenic acid (FUJIFILM Wako Pure Chemical Co., Osaka, Japan) and [^2^H_5_]-α-linolenic acid (Cayman Chemical Company, MI, United States), respectively, as previously described with slight modifications (Zimmerman and Feng, 1978; Kajiwara et al., 2012). Briefly, the extracted protein fraction of flaxseed acetone powder supplemented with recombinant *Oryza sativa* AOC (OsAOC) was used for enzymatic conversion. Glutathione-*S*-transferase (GST)-fused OsAOC was expressed in *Escherichia coli* Rosetta2 (DE3) (Merck, Darmstadt, Germany) according to the manufacturer’s instructions (Riemann et al., 2013), precipitated with ammonium sulfate and suspended in 50 mM potassium phosphate (pH 7.0). α-Linolenic acid and [^2^H_5_]-α-linolenic acid were suspended in 200 mM borate-NaOH buffer (pH 9.0) containing 5% (v/v) Tween 20, mixed with the extract of flaxseed acetone powder supplemented with recombinant GST-OsAOC, and incubated at 25°C for 150 min. The reaction was stopped by adding 1 N HCl to adjust the pH to 3.0–4.0, and the mixture was flushed with N_2_ gas for 30 min. The products were extracted with ethyl acetate, dried using an evaporator, and dissolved in methanol.

*iso*-OPDA was synthesized as shown in Scheme 1. All chemical reagents and solvents for chemical synthesis of *iso*-OPDA were obtained from Kanto Chemical Co., FUJIFILM Wako Pure Chemical Co., Nacalai Tesque Co., Tokyo Chemical Industry Co. (Tokyo, Japan), Sigma-Aldrich Co. (MO, United States), and GE Healthcare (IL, United States) and used without further purification. All anhydrous solvents were either dried by standard techniques and freshly distilled before use or purchased in anhydrous form and used as supplied.

^1^H and ^13^C NMR spectra were recorded on a JNM–ECS–400 spectrometer (JEOL, Tokyo, Japan) in deuterated chloroform using TMS as an internal standard. Fourier transform infrared (FT/IR) spectra were recorded on an FT/IR–4100 (JASCO, Tokyo, Japan). High–resolution (HR) electrospray ionization (ESI)–mass spectrometry (MS) analyses were conducted using a microTOF II (Bruker Daltonics Inc., MA, United States).

To a solution of THPO(CH_2_)_8_MgBr (0.53 M in THF, 5.3 mL, 2.81 mmol) was added a solution of 6 (Wang et al., 2021) (127 mg, 702 μmol) in THF (6.5 mL) at reflux temperature under argon atmosphere. After being stirred at 60°C for 2 h, the reaction mixture was allowed to cool to rt and 2 M HCl aq. (10 mL) was added. After 12 h of stirring, H_2_O was added and the water layer was extracted with EtOAc. The combined organic layers were washed with saturated aqueous NaCl, dried over Na_2_SO_4_ and concentrated under reduced pressure. After evaporation, the residue was purified by medium-pressure chromatography (Isolera, eluent: 40:60 *n*-hexane/EtOAc to EtOAc) to give the mixture contained **7** (73.2 mg). The mixture was carried on to the next step.

To a solution of the mixture (73.2 mg) in acetone (20 mL) was added Jones reagent (4.0 M solution, 2.5 mL, 10 mmol) at −20°C. After 1 h of stirring at −20°C, *i*-PrOH was added to quench the remaining reagent. Then, H_2_O (200 mL) was added and the water layer was extracted with EtOAc. The combined organic layers were washed with saturated aqueous NaCl, dried over Na_2_SO_4_ and concentrated under reduced pressure. The residue was purified by medium-pressure chromatography (Isolera, eluent: 0.5:50:50 AcOH/*n*-hexane/EtOAc to 0.5:99.5 AcOH/EtOAc) to give *iso*-OPDA (68.3 mg, 33% in two steps) as a colorless oil. ^1^H NMR (400 MHz, CDCl_3_); δ_*H*_ 5.37 (dtt, *J* = 10.6, 7.4, 1.6 Hz, 1H), 5.21 (dtt, *J* = 10.6, 7.0, 1.5 Hz, 1H), 2.93 (brd, *J* = 7.0 Hz, 2H), 2.52–2.45 (m, 2H), 2.42 (t, *J* = 7.4 Hz, 2 H), 2.39–2.32 (m, 4H), 2.15 (quintet, *J* = 7.4 Hz, 2H), 1.64 (brquintet, *J* = 7.5 Hz, 2H), 1.52 (brquintet, *J* = 7.5 Hz, 2H), 1.34 (brs. 6H), 0.99 (t, *J* = 7.4 Hz, 3H); ^13^C NMR (100 MHz, CDCl_3_); δ_C_ 209.49, 178.88, 174.35, 139.16, 132.19, 125.33, 34.18, 33.81, 31.27, 29.52, 29.16, 29.04, 28.89, 27.35, 24.59, 21.23, 20.60, 14.16; IR (film) cm^–1^: 3205, 2931, 2858, 1732, 1701; HRMS (ESI, positive) *m/z* [M + Na]^+^ Calcd. for C_18_H_28_O_3_: 2315.1936, found: 315.1927. The ^1^H and ^13^C NMR spectra of *iso*-OPDA are shown in Supplementary Figures 1, 2, respectively.

dn-OPDA and dn-*iso*-OPDA were prepared as previously described (Wang et al., 2021). [^2^H_5_]-dn-OPDA was purchased from Cayman Chemical Company.

### Chemical and Wounding Treatments

12-Oxo-phytodienoic acid, dn-OPDA, dn-*iso*-OPDA, JA, and JA-Ile were dissolved in dimethyl sulfoxide at a concentration of 10 mM and added to BCDATG liquid media to a final concentration of 50 μM, whereas [^2^H_5_]-OPDA, dissolved in 20% (v/v) aqueous dimethyl sulfoxide solution at a concentration of 1 mM, was added to BCDATG liquid media to 25 μM. *C. plumiforme* gametophores were incubated with media containing the chemicals for 2 h under continuous white light at 23°C.

For wounding treatment, *C. plumiforme* gametophores were cut by scissors into 5-mm long pieces, incubated under continuous white light at 23°C, and collected after 0, 0.5, 1, 2, and 4 h.

### 5*′*- and 3*′*-Rapid Amplification of cDNA Ends

To determine the nucleotide sequence of full-length *CpJAZ2* cDNA, total RNA was extracted from *C. plumiforme* gametophores 1 h after wounding using an RNeasy Plant Mini Kit (QIAGEN, Venlo, Netherlands). 5′- and 3′-RACE analyses of *CpJAZ2* were performed using the SMARTer RACE cDNA Amplification Kit (Clontech, CA, United States) and KOD FX (TOYOBO Co., Osaka, Japan) according to the manufacturer’s protocol. Amplified RACE products were cloned into pRACE using the In-Fusion HD Cloning Kit (Clontech) and sequenced to determine the full-length cDNA of *CpJAZ2*, which was amplified by end-to-end RT-PCR using KOD FX and a cDNA template prepared for 5′-RACE analysis. The full-length cDNA was cloned into the pCR-II-Blunt-TOPO vector (Thermo Fisher Scientific, MA, United States) to generate pCR-CpJAZ2, and the inserted fragment was sequenced. Sequences of primers used for RACE and cloning are provided in Supplementary Table 1.

### Cloning of COI1, JAZ, and MYC2 Genes

According to information from the genome database, coding sequences of *COI1*, *JAZ*, and *MYC2* genes of *C. plumiforme* were amplified by RT-PCR using KOD Plus Neo (TOYOBO Co.) and a cDNA template that was prepared for the 5′-RACE analysis. pCR-CpJAZ2 was used as a template for the amplification of *CpJAZ2*. Sequences of primers used for cloning are provided in Supplementary Table 1. Amplified fragments were cloned into pEU vectors (CellFree Sciences Co., Ehime, Japan), which are the expression vectors for a wheat germ cell-free protein expression system, using the In-Fusion HD Cloning Kit (Clontech) or SLiCE (Motohashi, 2015). *COI1* and *MYC2* genes were cloned into pEU-E01-GST-PS-MCS-N1, and *JAZ* genes were cloned into pEU-E01-DYKDDDDK-MCS-N1. The inserted fragments were sequenced.

### Sequence Identification and Phylogenetic Analyses

Transcriptome data of *C. plumiforme* treated with CuCl_2_, which induces oxidative stress and defense responses in plants, were available based on our previous RNA-seq analyses, the results of which are deposited in the DDBJ Sequence Read Archive under accession no. DRA010138 (Okada et al., 2016). The *C. plumiforme* genome sequence and annotation for estimated coding proteins are deposited in the Genome Sequence Archive under accession no. PRJCA001833 (Mao et al., 2020). *C. plumiforme* genes homologous to *COI1*, *JAZ*, and *MYC* were obtained using the corresponding genes in *M. polymorpha* as a query for a BLAST search using transcriptome data and the genome sequence.

Phylogenetic and molecular evolutionary analyses were conducted using MEGA version X (Kumar et al., 2018) with the neighbor-joining method (Saitou and Nei, 1987). Sequence alignments were constructed in MEGA using Muscle (Edgar, 2004). Neighbor-joining trees were also constructed. Bootstrap values were evaluated with 1,000 replications. Sequence alignments were performed using the BioEdit software version 7.2.5.

### Statistical Analysis

One-way ANOVA with Tukey’s *post hoc* test was performed using Excel (Microsoft Co., WA, United States) and Statcel 4 (OMS Publishing Inc., Saitama, Japan) software.

### Quantification of OPDAs, JA, and JA-Ile

Wounded *C. plumiforme* gametophores (approximately 50 mg fresh weight) were homogenized and suspended in 2 mL of 80% (v/v) aqueous methanol, and [^2^H_5_]-OPDA (2.5 ng), [^2^H_5_]-JA (10 ng), [^13^C_6_]-JA-Ile (10 ng), and [^2^H_5_]-dn-OPDA (10 ng) were added as internal standards. The extracts were purified and concentrated as previously described (Miyamoto et al., 2016). [^2^H_5_]-OPDA-fed gametophores (approximately 7 mg fresh weight) were homogenized and purified using the same method without internal standards. The resultant samples were subjected to liquid chromatography with electrospray ionization tandem mass spectrometry (LC-ESI-MS/MS) composed of a quadrupole tandem mass spectrometer (Agilent 6460 Triple Quadrupole mass spectrometer) with an electrospray ion source and an Agilent 1200 separation module. OPDAs, JA, and JA-Ile were quantified as previously described, with slight modifications (Tamiru et al., 2016). The mobile phase consisted of 0.05% acetic acid in water (solvent A) and 0.05% acetic acid in acetonitrile (solvent B). Elution was conducted using a linear gradient of solvent B from 3 to 70% over 15 min and 70 to 98% over 5 min at a flow rate of 0.2 mL/min. The retention times of OPDA, *iso*-OPDA, dn-OPDA, dn-*iso*-OPDA, JA, and JA-Ile were 17.1, 16.8, 15.9, 15.5, 13.4, and 15.0 min, respectively. The precursor-to-product transitions monitored were *m*/*z* 291/165 for OPDA and *iso*-OPDA, *m*/*z* 296/170 for [^2^H_5_]-OPDA and [^2^H_5_]-*iso*-OPDA, *m*/*z* 263/165 for dn-OPDA and dn-*iso*-OPDA, *m*/*z* 268/170 for [^2^H_5_]-dn-OPDA and [^2^H_5_]-dn-*iso*-OPDA, m/z 209/59 for JA; *m*/*z* 214/62 for [^2^H_5_]-JA, *m*/*z* 322/130 for JA-Ile, and *m*/*z* 328/136 for [^13^C_6_]-JA-Ile. The concentrations of OPDAs and JA in the wounded samples were calculated based on the relative proportions of the peak area relative to the internal standards, whereas those of OPDAs in the [^2^H_5_]-OPDA-fed samples were determined based on the concentration of the authentic standard run under the same analysis conditions.

### Gene Expression Analysis

Total RNA was extracted from *C. plumiforme* gametophores using an RNeasy Plant Mini Kit (QIAGEN) and subjected to cDNA synthesis using a PrimeScript RT Reagent Kit with gDNA Eraser (Takara Bio, Shiga, Japan). Quantitative RT-PCR (qRT-PCR) was performed using a THUNDERBIRD SYBR qPCR Mix (TOYOBO Co.) on an ABI 7500 Fast Real-Time PCR System (Applied Biosystems, CA, United States). The ddCt method was used to calculate transcript levels. *CpACT3* (DDBJ ID: LC129863) was used as an internal control to normalize the amount of mRNA. For each sample, the mean value from biological triplicate samples was used to calculate the transcript abundance. Sequences of primers used for qRT-PCR analysis are provided in Supplementary Table 1.

### *In vitro* Cell-Free Protein Expression

GST- or FLAG tag-fused CpMYC2 and CpJAZ proteins were expressed using a WEPRO7240 Expression Kit (CellFree Sciences Co.), which is a wheat germ cell-free protein expression system, according to the manufacturer’s protocol. Expressed samples were centrifuged at 17,800 × *g* for 5 min at 4°C, and the supernatants were used for subsequent analyses. FLAG-CpJAZ2 was diluted five times before use because its expression was higher than that of FLAG-CpJAZ1 and FLAG-CpJAZ3. A portion of the supernatant was mixed with Laemmli SDS sample buffer and boiled at 96°C for 10 min; the boiled proteins were subjected to protein gel blot analysis. The remaining supernatants were subjected to co-immunoprecipitation analysis.

### Co-immunoprecipitation Analysis

For each sample, 10 μL of the supernatant was used. GST-CpMYC2a, GST-CpMYC2b, and GST were mixed with FLAG-CpJAZ1, FLAG-CpJAZ2, or FLAG-CpJAZ3 in IP buffer [25 mM Tris pH 7.8, 50 mM NaCl, 5% (v/v) glycerol, 20 mM 2-mercaptoethanol, and 0.05% (v/v) Tween 20] in the presence of a cOmplete EDTA-free protease inhibitor cocktail (Merck). After incubation at 4°C for 60 min, 0.2 μg of anti-FLAG M2 monoclonal antibody (Merck) was added to each sample. The samples were further incubated at 4°C for 60 min with rotation, and SureBeads Protein G Magnetic Beads (Bio-Rad, CA, United States) were added. Finally, after incubation at 4°C for 60 min with rotation, the beads were washed three times with PBS containing 0.1% (v/v) Tween 20. The resultant beads were boiled in double-diluted sample buffer solution (2ME +) (× 2) (Fujifilm Co.) containing 100 mM dithiothreitol at 60°C for 10 min. The boiled proteins were subjected to protein gel blot analysis.

### Protein Gel Blot Analysis

The boiled samples were subjected to SDS-PAGE on 10% (w/v) polyacrylamide gels and transferred to a nitrocellulose membrane (Bio-Rad). GST-CpMYC2a, GST-CpMYC2b, and GST were detected using an anti-GST HRP conjugate [dilution 1:5,000 (v/v)] (Cytiva, Tokyo, Japan) and iBind Flex Western Device (Thermo Fisher Scientific), whereas FLAG-CpJAZ1, FLAG-CpJAZ2, and FLAG-CpJAZ3 were detected using anti-FLAG M2 monoclonal antibody (Merck) [dilution 1:1,000 (v/v)] as the primary antibody and anti-mouse IgG, HRP-linked whole antibody sheep [dilution 1:10,000 (v/v)] (Cytiva) as the secondary antibody with an iBind Flex Western Device. Chemiluminescent detection was carried out using Immobilon Western Chemiluminescent HRP Substrate (Merck) and a ChemiDoc imaging system (Bio-Rad).

## Results

### Genomic Mining of COI1, JAZ, and MYC2 Gene Homologs in *Calohypnum plumiforme*

In the process of elucidating the molecular mechanism that induces momilactone production in *C. plumiforme*, we previously reported that OPDA induces momilactone accumulation, suggesting that OPDA or its derivatives act as signaling molecules (Okada et al., 2016). The OPDA/dn-OPDA signaling pathway has been identified in the bryophyte *M. polymorpha*. Therefore, we first searched for genes homologous to *COI1*, *JAZ*, and *MYC* using the corresponding genes in *M. polymorpha* as a query using transcriptome data and the genome sequence of *C. plumiforme*.

#### COI1 in *Calohypnum plumiforme*

We found two genes similar to *MpCOI1* (Monte et al., 2018) from transcriptome data, and these genes were also found in the *C. plumiforme* genome database. These genes were designated as *CpCOI1a* and *CpCOI1b* (Table 1). We cloned the coding sequences of *CpCOI1a* and *CpCOI1b* based on the information from the genome annotation, which were identical to annotated sequences in the genome database and deposited in GenBank under accession no. MW775560 (*CpCOI1a*) and MW775561 (*CpCOI1b*).

**TABLE 1:** *COI1*, *JAZ*, and *MYC2* genes in *C. plumiforme*.

Gene name	ID in genome sequence archive	GenBank accession number
*CpCOI1a*	GWHTAMMO020093	MW775560
*CpCOI1b*	GWHTAMMO007259	MW775561
*CpJAZ1*	GWHTAMMO005348	MW775562
*CpJAZ2*	Not annotated	MW775563
*CpJAZ3*	GWHTAMMO025074	MW775564
*CpMYC2a*	GWHTAMMO011051	MW775565
*CpMYC2b*	GWHTAMMO007338	MW775566

**FIGURE 1:**
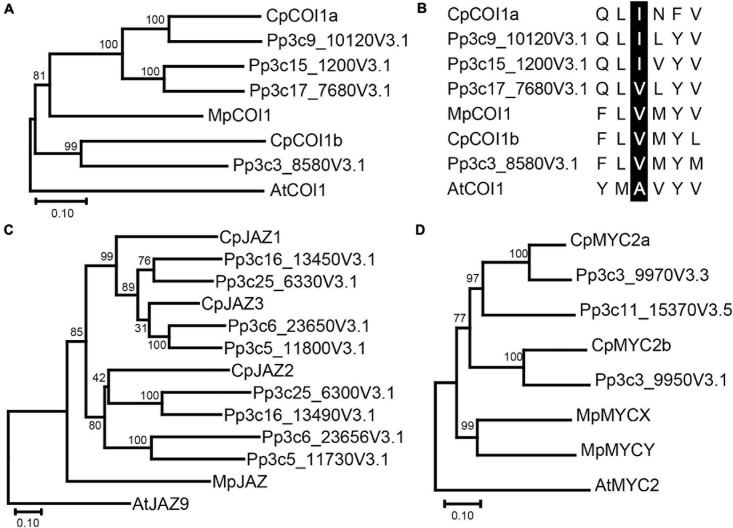
Phylogenetic analysis of COI1 **(A)**, JAZ **(C)**, MYC2 **(D)** homologs, and the residue essential for dn-OPDA and dn-*iso*-OPDA recognition **(B)**. **(A)** Phylogenetic trees (amino acid sequences) were obtained using the neighbor-joining method as described in the “Materials and Methods.” The percentage of replicate trees in which the associated taxa clustered together in the bootstrap test (1000 replicates) is shown next to the branches. COI1 homologs in *M*. *polymorpha* and *P. patens* are also included. The phylogenetic tree was rooted in AtCOI1. Gene IDs are as follows: MpCOI1: Mapoly0025s0025 and AtCOI1: AT2G39940. **(B)** The amino acid sequence of the valine residue at position 377 (V377) of MpCOI1 and its surroundings is shown with the corresponding sequences of other COI1 homologs. V377 in MpCOI1 and its corresponding residues in other COI1 homologs are highlighted by black. **(C)** Phylogenetic trees (amino acid sequences) were obtained using the neighbor-joining method as described in the “Materials and Methods.” The percentage of replicate trees in which the associated taxa clustered together in the bootstrap test (1000 replicates) is shown next to the branches. JAZ homologs in *M*. *polymorpha* and *P. patens* are also included. The phylogenetic tree was rooted in AtJAZ9. Gene IDs are as follows: MpJAZ: Mapoly0097s0021 and AtJAZ9: AT1G70700. **(D)** Phylogenetic trees (amino acid sequences) were obtained using the neighbor-joining method as described in the “Materials and Methods.” The percentage of replicate trees in which the associated taxa clustered together in the bootstrap test (1,000 replicates) is shown next to the branches. MYC2 homologs in *M*. *polymorpha* and *P. patens* are also included. The phylogenetic tree was rooted in AtMYC2. Gene IDs are as follows: MpMYCX: Mapoly0018s0019; MpMYCY: MapolyY_B0018; and AtMYC2: AT1G32640.

Phylogenetic analysis was conducted using the amino acid sequences of COI1 in *C. plumiforme*, *M. polymorpha*, and *P. patens* with *Arabidopsis thaliana* COI1 as an outgroup. CpCOI1a and three sequences from *P. patens*, were grouped in the same clade as MpCOI1 (Figure 1A), suggesting that these COI1s are conserved in both mosses and liverworts. However, CpCOI1b and COI1 of *P. patens* formed another clade (Figure 1A), suggesting that these two COI1s are moss-specific. The alignment of these sequences revealed that the residues involved in the recognition of ligands or the interaction with JAZ proteins reported by Sheard et al. (2010) were conserved in COI1 of *C. plumiforme* (Supplementary Figure 3). Monte et al. (2018) reported that valine at position 377 in MpCOI1 is essential for the recognition of dn-OPDA and dn-*iso*-OPDA and that this residue is substituted with alanine on COI1s recognizing a JA-Ile such as AtCOI1. COIs in the mosses predominantly show isoleucine in this position, which is a hydrophobic residue similar to valine (Monte et al., 2018). Consistent with this, the isoleucine residue is conserved in CpCOI1a, although the corresponding residue in CpCOI1b is valine (Figure 1B).

#### JAZ in *Calohypnum plumiforme*

**FIGURE 2:**
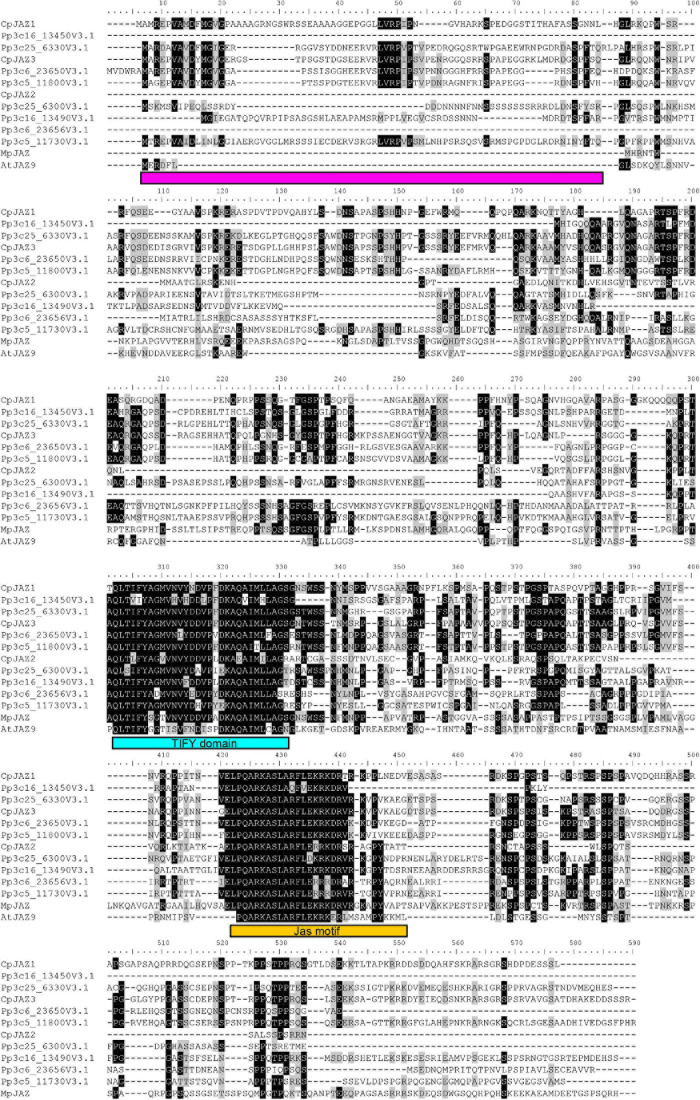
Sequence alignment of JAZ proteins. Sequences of JAZ homologs were aligned using MEGA version X using Muscle. Sequence alignments were drawn using the BioEdit software version 7.2.5. The shading thresholds for identical or similar residues were 40%. Identical and similar residues are highlighted by black and gray, respectively. The TIFY domain and Jas motifs are indicated by blue and orange squares, respectively. The conserved sequence in the N-terminus of the moss JAZs is indicated by magenta squares.

We found three genes similar to *MpJAZ* (Monte et al., 2019) from transcriptome data designated as *CpJAZ1, CpJAZ2*, and *CpJAZ3* (Table 1). *CpJAZ1* and *CpJAZ3* were also annotated in the *C. plumiforme* genome database. We cloned the coding sequences of *CpJAZ1* and *CpJAZ3* according to the information from the genome database, which were identical to annotated sequences in the genome database and deposited in GenBank under accession no. MW775562 (*CpJAZ1*) and MW775564 (*CpJAZ3*). *CpJAZ2* was not annotated in the genome database, and only a partial sequence of *CpJAZ2* was obtained from the RNA-seq data. Therefore, we performed 5′- and 3′- RACE to obtain the full-length sequence of *CpJAZ2*, which was deposited in GenBank under accession no. MW775563. Phylogenetic analysis was conducted using the amino acid sequences of JAZ in *C. plumiforme*, *M. polymorpha*, and *P. patens*, with *A*. *thaliana* JAZ9 as an outgroup. CpJAZ1 and CpJAZ3, along with four sequences from *P. patens*, were grouped in the same clade, whereas CpJAZ2 and four sequences of *P. patens* formed another clade (Figure 1C), suggesting that JAZ proteins evolved into two groups in mosses. The alignment of these sequences revealed that the Jas motif and TIFY domain were conserved in CpJAZ1, CpJAZ2, and CpJAZ3 (Figure 2). N-terminal sequences were conserved among several JAZs in *C. plumiforme* and *P. patens*, including CpJAZ1 and CpJAZ3. This conserved sequence was not found in MpJAZ or AtJAZ9 (Figure 2).

#### MYC2 in *Calohypnum plumiforme*

We found two genes similar to the *M. polymorpha* MYC2 ortholog gene, *MpMYCY* (Peñuelas et al., 2019), from transcriptome data, and these genes were also found in the *C. plumiforme* genome database. These two genes were designated as *CpMYC2a*, and *CpMYC2b* (Table 1). We cloned the coding sequences of *CpMYC2a* and *CpMYC2b* according to the information from the genome database; that of *CpMYC2a* had a 12 bp substitution with the annotation of the genome database, possibly due to misassembly of the genome sequence or polymorphism among the *C. plumiforme* lines used in genome sequencing and our experiments (Supplementary Figure 4A), whereas that of *CpMYC2b* lacked a 99 bp sequence that was annotated as exons in the genome database. This sequence had a putative 3′-splice site (5′-AG-3′) and branchpoint sequence (5′-CTNAN-3′; Brown et al., 1996), suggesting that the longer region was removed in the *C. plumiforme* gametophores used in the present study (Supplementary Figure 4B). The cloned coding sequences were deposited in GenBank under accession no. MW775565 (*CpMYC2a*) and MW775566 (*CpMYC2b*) and used for subsequent analyses.

Phylogenetic analysis was conducted using the amino acid sequences of MYC2 in *C. plumiforme*, *M. polymorpha*, and *P. patens* with *A*. *thaliana* MYC2 as an outgroup. CpMYC2a and two sequences from *P. patens* were grouped in the same clade, whereas CpMYC2b and one sequence from *P. patens* formed another clade (Figure 1D), suggesting that MYC2 proteins evolved into two groups in mosses. The alignment of these sequences revealed that the JID and bHLH domains were conserved in CpMYC2a and CpMYC2b (Supplementary Figure 5).

### Quantification of Endogenous Levels of OPDA, dn-OPDA, and Their Isomers

**FIGURE 3:**
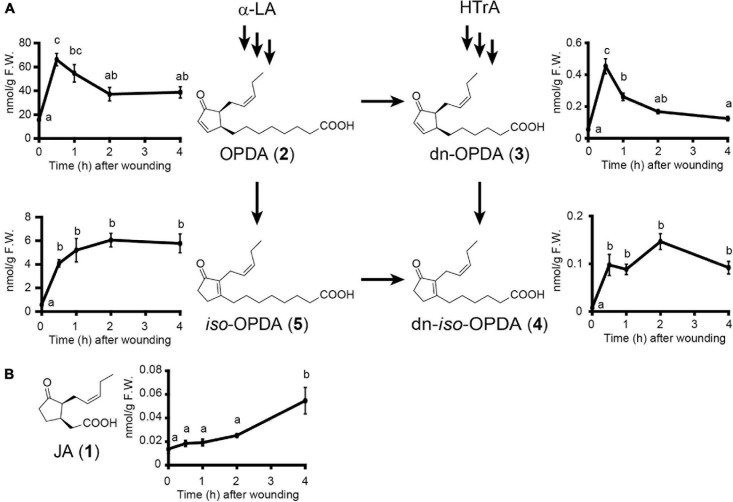
Effects of wounding on accumulation levels of endogenous OPDA, *iso*-OPDA, dn-OPDA, dn-*iso*-OPDA **(A)**, and JA **(B)**. **(A)** Values indicate the amount of OPDA, *iso*-OPDA, dn-OPDA, and dn-*iso*-OPDA in *C. plumiforme* gametophores after wounding (*n* = 3). **(B)** Values indicate the amount of JA in *C. plumiforme* gametophores after wounding (*n* = 3). Arrows indicate the proposed biosynthetic pathways in *M*. *polymorpha.* Bars indicate standard errors of the means. Statistically different data groups are indicated with different letters (*p* < 0.05, one-way ANOVA with Tukey’s *post hoc* test). LA, linolenic acid; HTrA, hexadecatrienoic acid.

We found putative OPDA/dn-OPDA signaling components in *C. plumiforme*; however, the endogenous levels of OPDA, dn-OPDA, and their isomers, which accumulate in gametophores after wounding, have not yet been investigated. Therefore, we measured these. The levels of OPDA and dn-OPDA increased and peaked at 30 min after wounding and then decreased (Figure 3). *iso*-OPDA and dn-*iso*-OPDA also accumulated after 30 min but remained at a high level for 4 h after wounding. After wounding, the level of OPDA was highest, followed by that of *iso*-OPDA, which was approximately one-tenth of that of OPDA. The amounts of dn-OPDA and dn-*iso*-OPDA were less than one-hundredth that of OPDA. We detected a small amount of JA, although this was below the detection limit of a previous study (Okada et al., 2016). Additionally, the level of JA 4 h after wounding was significantly higher than that in unwounded *C. plumiforme* gametophores (Figure 3). The amount of JA was about 1/1000 of that of OPDA, and it was almost consistent with a steady-state level of JA in angiosperms, such as *A. thaliana* and rice (Park et al., 2002; Riemann et al., 2013). JA-Ile, which is a biologically active form in seed plants, was not detected with or without wounding.

### *In planta* Conversion of Labeled-OPDA to *iso*-OPDA, dn-OPDA, and dn-*iso*-OPDA

**FIGURE 4:**
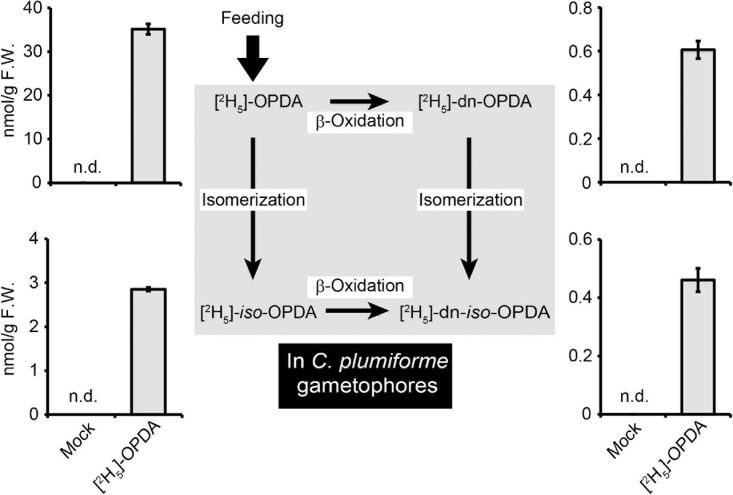
Conversion of deuterium-labeled OPDA [(^2^H_5_)-OPDA] in *C. plumiforme* gametophores. The values indicate the amount of [^2^H_5_]-OPDA, *iso*-OPDA, dn-OPDA, and dn-*iso*-OPDA in *C. plumiforme* gametophores after [^2^H_5_]-OPDA treatment at 25 μM (*n* = 3). Mock samples were treated with BCDATG liquid media containing 0.5% (v/v) dimethyl sulfoxide as a negative control. The arrows indicate the proposed biosynthetic pathways in *M*. *polymorpha.*

Two synthetic pathways for dn-OPDA and dn-*iso*-OPDA are shown in *M. polymorpha*. One is the conversion of OPDA to dn-OPDA and dn-*iso*-OPDA; the other is the direct synthesis of dn-OPDA and dn-*iso*-OPDA from hexadecatrienoic acid, but not *via* OPDA (Monte et al., 2018). To investigate the conversion of OPDA to dn-OPDA and dn-*iso*-OPDA, we fed 25 μM deuterium-labeled OPDA [(^2^H_5_)-OPDA] to *C. plumiforme* gametophores and then measured the level of deuterium-labeled derivatives by LC-MS/MS. Both [^2^H_5_]-dn-OPDA and [^2^H_5_]-dn-*iso*-OPDA accumulated after [^2^H_5_]-OPDA treatment, indicating that OPDA can be efficiently converted to dn-OPDA in *C. plumiforme* (Figure 4). Isomerization of OPDA to *iso*-OPDA was also observed because [^2^H_5_]-*iso*-OPDA also accumulated after [^2^H_5_]-OPDA treatment (Figure 4). The ratio of the amounts of [^2^H_5_]-OPDA, [^2^H_5_]-*iso*-OPDA, [^2^H_5_]-dn-OPDA, and [^2^H_5_]-dn-*iso*-OPDA in [^2^H_5_]-OPDA-treated *C. plumiforme* was similar to that of the unlabeled compounds in wounded plants.

### OPDA, dn-OPDA, and dn-*iso*-OPDA Induce the Expression of CpJAZ Genes

**FIGURE 5:**
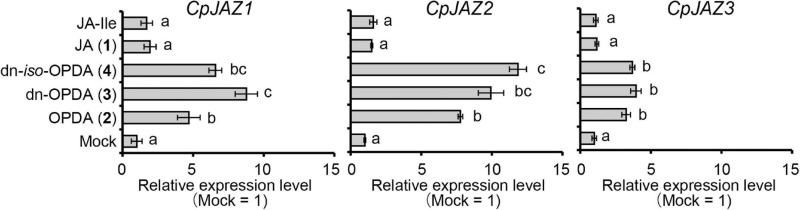
Effect of wounding on the expression of *CpJAZ1*, *CpJAZ2*, and *CpJAZ3* as determined by qRT-PCR. Values indicate relative expression levels after treatment with OPDA, dn-OPDA, dn-*iso*-OPDA, JA, and JA-Ile at 50 μM (*n* = 3). Mock samples were treated with BCDATG liquid media containing 0.5% (v/v) dimethyl sulfoxide as a negative control. Expression levels were normalized to the expression of *CpACT3*, and the bars indicate the standard errors of the means. Statistically different data groups are indicated with different letters (*p* < 0.05, one-way ANOVA with Tukey’s *post hoc* test).

The expression of *JAZ* genes encoding repressors is induced by the ligand for COI1 to repress the signal (Chini et al., 2007; Thines et al., 2007; Monte et al., 2019). We examined the expression of *JAZ* genes after OPDA, dn-OPDA, or dn-*iso*-OPDA treatment in *C. plumiforme*. As shown in Figure 5, the expression of *CpJAZ1*, *CpJAZ2*, and *CpJAZ3* was induced by treatment with OPDA, dn-OPDA, or dn-*iso*-OPDA. JA and JA-Ile did not affect the expression of these genes. These results suggest that OPDA and/or both isomers of dn-OPDA are biologically active. dn-OPDA and dn-*iso*-OPDA might be recognized by CpCOI1 as in *M. polymorpha*, although there remains the possibility that another metabolite from OPDA and dn-OPDA acts as a ligand of CpCOI1.

### Physical Interaction Between CpJAZs and CpMYC2s

**FIGURE 6:**
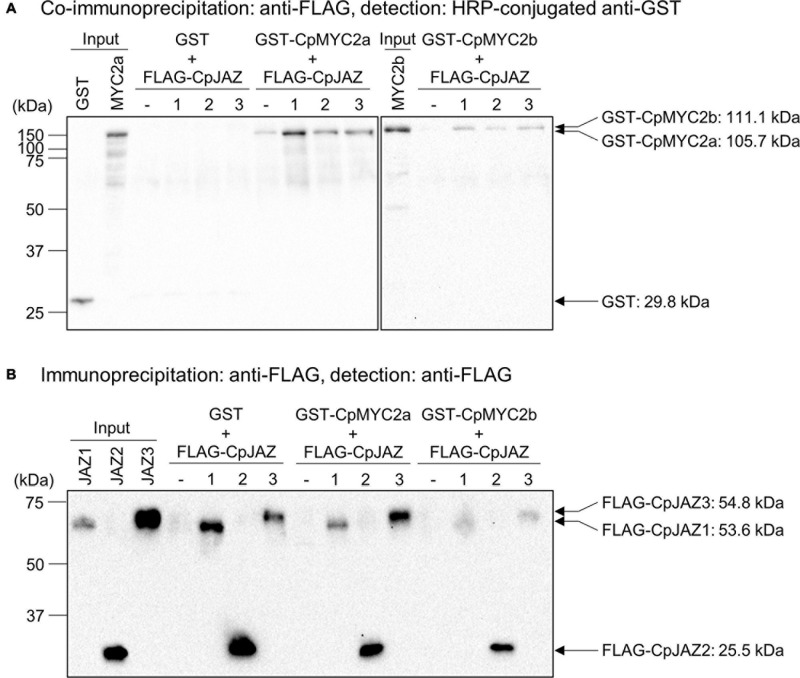
Co-immunoprecipitation analysis investigating the interaction between CpJAZs and CpMYC2s. **(A)** Proteins co-immunoprecipitated by anti-FLAG antibodies were analyzed. GST and GST-fused proteins were detected by protein gel blot analysis using a horseradish peroxidase-conjugated anti-GST antibody. **(B)** Proteins immunoprecipitated by anti-FLAG antibody were analyzed. FLAG-tagged proteins were detected by protein gel blot analysis using an anti-FLAG antibody.

JAZ proteins physically interact with MYC2, MYC3 and MYC4 transcription factors to repress downstream signaling (Chini et al., 2007; Thines et al., 2007; Fernández-Calvo et al., 2011; Peñuelas et al., 2019). To investigate the physical interaction between JAZ and MYC2 proteins from *C. plumiforme*, we performed co-immunoprecipitation analyses. GST- or FLAG tag-fused CpMYC2 and CpJAZ proteins were expressed using a wheat germ cell-free protein expression system and detected by protein gel blot analysis at a position consistent with the predicted molecular weight; GST: 29.3 kDa, GST-CpMYC2a: 105.7 kDa, GST-CpMYC2b: 111.1 kDa, FLAG-CpJAZ1: 53.6 kDa, FLAG-CpJAZ2: 25.5 kDa, and FLAG-CpJAZ3: 54.8 kDa (Supplementary Figure 6). Expressed MYC2 and JAZ proteins were mixed and then immunoprecipitated using an anti-FLAG antibody. Immunoprecipitated proteins were analyzed by protein gel blot analysis using anti-GST or anti-FLAG antibodies. GST-CpMYC2a and CpMYC2b, but not GST, were detected after immunoprecipitation with CpJAZ1, CpJAZ2, or CpJAZ3 (Figure 6A). Additionally, GST-CpMYC2a and CpMYC2b in immunoprecipitated proteins with CpJAZs were more strongly detected than those without FLAG-tagged proteins. Immunoprecipitated FLAG-CpJAZs were detected using an anti-FLAG antibody (Figure 6B). These results indicate that CpJAZ1, CpJAZ2, and CpJAZ3 directly interact with CpMYC2a or CpMYC2b *in vitro*.

## Discussion

### COI1, JAZ, and MYC2 Genes Were Conserved in *Calohypnum plumiforme*

In this study, we investigated signaling mechanisms promoted by OPDA and its derivatives in *C. plumiforme*. We found that COI, JAZ, and MYC2 sequences, including domains important for their functions, were conserved (Figures 1, 2 and Supplementary Figures 3, 5). Moreover, in CpCOI1a and CpCOI1b, a hydrophobic residue that is important for the recognition of dn-OPDA and dn-*iso*-OPDA was conserved (Figure 1B), suggesting that CpCOI1a and CpCOI1b recognize dn-OPDA and its related derivatives.

CpJAZ1, CpJAZ2, and CpJAZ3 conserved the Jas motif and TIFY domain as MpJAZ and AtJAZ9, implying that JAZs of *C. plumiforme* have similar functions to repress the downstream signaling of COI1 and MYCs. Several JAZs from mosses had conserved N-terminal sequences, which were not found in MpJAZ and AtJAZ9 (Figure 2). These sequences might be involved in the specific function of JAZs in mosses, although we could not find a similar sequence with identifiable functions by the BLAST search. Although the liverwort *M*. *polymorpha* has a single *JAZ* gene, mosses *C. plumiforme* and *P. patens* have at least three and eight *JAZ* genes, respectively. The number of JAZs in seed plants is even higher; for example, there are 13 and 15 JAZs in *Arabidopsis* and rice, respectively (Pauwels and Goossens, 2011; Chini et al., 2016; Uji et al., 2016). Genome duplication has been reported in mosses (Rensing et al., 2007; Gao et al., 2021), which may explain why there are more JAZs in mosses than in liverworts.

As *C. plumiforme* possessed fewer *COI1*, *JAZ*, and *MYC2* genes than *P. patens* (Figure 1), less redundancy is expected. Unfortunately, we could not use reverse genetics approaches because of the absence of a transformation system in *C. plumiforme*. Because of the small number of COI1, JAZ, and MYC2 homologs and their importance in the research on the evolution of plant chemical defenses, the development of a transformation system for *C. plumiforme* is needed.

### OPDA, dn-OPDA, and Their Isomers Accumulated After Wounding Treatment in *Calohypnum plumiforme*

To determine a biologically active molecule in OPDA signaling in *C. plumiforme*, we performed quantification analyses of OPDA, dn-OPDA, and their isomers. We observed that OPDA, *iso*-OPDA, dn-OPDA, and dn-*iso*-OPDA accumulated after wounding (Figure 3). A small amount of JA was detected and increased by wounding, although JA-Ile was not detected (Figure 3). Bryophytes do not have OPR3 orthologs, which catalyze the reduction of OPDA, but homologous genes to *AtOPR2*, which catalyzes 4,5-didehydro-JA reduction to produce JA (Han, 2016; Chini et al., 2018; Wasternack and Hause, 2018). Consistent with these reports, genes homologous to AtOPR2 were found in the *C. plumiforme* genome. These translation products possibly catalyze the reduction of OPDA homologs, such as dn-OPDA. However, the contribution of OPR should be small in *C. plumiforme*, since the level of JA is very low.

We conducted a feeding experiment using [^2^H_5_]-OPDA to investigate whether *iso*-OPDA, dn-OPDA, and dn-*iso*-OPDA were synthesized from OPDA. [^2^H_5_]-dn-OPDA was detected after the feeding of [^2^H_5_]-OPDA (Figure 4), indicating that OPDA was converted to dn-OPDA by β-oxidation in *C. plumiforme* as reported in *M*. *polymorpha* (Monte et al., 2018). The detection of [^2^H_5_]-*iso*-OPDA in [^2^H_5_]-OPDA-fed plants revealed that the isomerization of OPDA occurred (Figure 4). [^2^H_5_]-dn-*iso*-OPDA was observed after feeding [^2^H_5_]-OPDA (Figure 4). dn-*iso*-OPDA may be synthesized from OPDA by the β-oxidation of *iso*-OPDA and/or isomerization of dn-OPDA as proposed in *M*. *polymorpha* and *P. patens* (Monte et al., 2018; Mukhtarova et al., 2020). In *M*. *polymorpha*, dn-OPDA is synthesized from hexadecatrienoic acid, not *via* OPDA, in addition to the pathway from OPDA. It is unknown whether a biosynthetic pathway from hexadecatrienoic acid exists in *C. plumiforme*. The ratio of [^2^H_5_]-OPDA, [^2^H_5_]-*iso*-OPDA, [^2^H_5_]-dn-OPDA, and [^2^H_5_]-dn-*iso*-OPDA in [^2^H_5_]-OPDA-treated plants was approximately 60:5:1:1, which is similar to that of the corresponding endogenous compounds after wounding. Therefore, the pathway for the synthesis of dn-OPDA from OPDA may also be functional in *C. plumiforme* after wounding. However, the metabolic flow of the β-oxidation of OPDA and/or *iso*-OPDA seems to be moderate compared with that in *M. polymorpha*.

### OPDA, dn-OPDA, and dn-*iso*-OPDA Induce CpJAZ Gene Expression

We investigated the inductive activity of OPDA, dn-OPDA, and dn-*iso*-OPDA on the expression of *JAZ* genes. Although they induced the expression of *CpJAZ1*, *CpJAZ2*, and *CpJAZ3*, JA and JA-Ile did not (Figure 5). We attempted to reveal the ligand for CpCOI1a and CpCOI1b by analyzing the physical interaction between COI1-JAZ using co-immunoprecipitation and yeast two-hybrid assays in the presence of OPDA, dn-OPDA, or dn-*iso*-OPDA, but we could not prove this interaction. This may be because the expressed COI1 protein did not retain its activity or another molecule is the ligand for COI1 of *C. plumiforme*.

### Difference in OPDA Profiles of Bryophytes

We showed that OPDA was the most abundant in wounded *C. plumiforme*, followed by *iso*-OPDA, dn-OPDA, and dn-*iso*-OPDA (Figure 3). In the model liverwort *M*. *polymorpha*, dn-*iso*-OPDA is most abundant after wounding, followed by OPDA and dn-OPDA, the levels of which are almost comparable (Monte et al., 2018). Additionally, in the model moss *P. patens*, *iso*-OPDA is predominant, and smaller amounts of OPDA and dn-*iso*-OPDA exist with or without the feeding of α-linolenic acid (Mukhtarova et al., 2020). The differences in the profiles of OPDA and its derivatives among the three species indicate that the metabolic rates of isomerization and β-oxidation are different. The biological significance of the different profiles of OPDAs requires further study.

### CpJAZs Interact With CpMYC2s

JAZ proteins interact with MYC2, MYC3 and MYC4 and repress downstream genes in various plants (Chini et al., 2007; Thines et al., 2007; Fernández-Calvo et al., 2011; Wasternack and Hause, 2013; Peñuelas et al., 2019). Co-immunoprecipitation analyses revealed that CpJAZ1, CpJAZ2, and CpJAZ3 physically interacted with CpMYC2a or CpMYC2b (Figure 6). Although the target genes of CpMYC2s are still unknown, CpJAZs might repress the transcription of these target genes at a steady state. Because a histidine residue in the bHLH domain that determines which DNA sequence to bind was conserved in CpMYC2s (Supplementary Figure 5), we assumed that CpMYC2s might bind to a G-box motif (CACGTG), similar to AtMYC2 and MpMYCs (Chini et al., 2007; Peñuelas et al., 2019). We found a G-box motif approximately 60 and 100 bp upstream of the transcription start site of *CpJAZ1* and *CpJAZ2*, respectively. There is no G-box motif in the 2,000 bp upstream region from the transcription start site of *CpJAZ3*, but a G-box motif exists in the 5′- untranslated region of *CpJAZ3*. CpMYC2a and CpMYC2b possibly bind to these G-box motifs and regulate the inductive expression of *CpJAZ* genes in response to OPDA or its derivatives.

### Conclusion

We found that OPDA, dn-OPDA, and their isomers accumulated in *C. plumiforme* after wounding at different abundance ratios than in *M*. *polymorpha* and *P. patens*, and that OPDA, dn-OPDA, and dn-*iso*-OPDA induced *CpJAZ* gene expression, indicating that they all have biological activity in *C. plumiforme*. *COI1*, *JAZ*, and *MYC2* genes were conserved, as were the physical interactions between JAZ and MYC2 proteins. These results indicate that COI1-JAZ-MYC mediated signaling is conserved. At this time, the ligand(s) of CpCOI1s remain unknown. Future research using genetic approaches and/or other biochemical methods will reveal the interaction between CpCOI1s and CpJAZs and the ligand(s) of CpCOI1.

## Data Availability Statement

The datasets presented in this study can be found in online repositories. The names of the repository/repositories and accession number(s) are as follows: https://www.ncbi.nlm.nih.gov/genbank/, MW775560; MW775561; MW775562; MW775563; MW775564; MW775565; MW775566.

## Author Contributions

KMi and KO designed the study. HI, NA, KMu, KS, SM, and MT performed the biological experiments. EY carried out LC/MS/MS analyses. KU synthesized (–)-JA and JA-Ile. NK and MU synthesized dn-OPDA, dn-*iso*-OPDA, and *iso*-OPDA. YH, TSh, and IG synthesized OPDA and [^2^H_5_]-OPDA. TK, YT, and MU performed the protein-protein interaction analyses. AN and TSa expressed JAZ proteins. HI and KMi performed all the computational analyses. KMi, HN, and KO supervised the experiments. KMi and KO wrote the manuscript. HI, NK, TSh, and IG revised the manuscript. All authors read and approved the final manuscript.

## Conflict of Interest

The authors declare that the research was conducted in the absence of any commercial or financial relationships that could be construed as a potential conflict of interest.

## Supplementary Material

Supplementary Figure 1 ^1^H NMR spectrum of *iso*-OPDA.

Supplementary Figure 2 ^13^C NMR spectrum of *iso*-OPDA.

Supplementary Figure 3 Sequence alignment of COIs.

Supplementary Figure 4 Differences between cloned CpMYC2s sequences and annotated sequences in the genome database.

Supplementary Figure 5 Sequence alignment of MYC2.

Supplementary Figure 6 Expression of GST-CpMYC2s and FLAG-JAZs.

Supplementary Table 1 Primers used in this study.
