# Supplementary material for: Deciphering OPDA Signaling Components in the Momilactone-Producing Moss Calohypnum plumiforme
Source: Front Plant Sci. 2021 May 31;12:688565. doi: 10.3389/fpls.2021.688565 (PMC8201998; doi:10.3389/fpls.2021.688565)
Supplement: Supplementary Figure 1 — 1H NMR spectrum of iso-OPDA. [file Image_1.PDF]

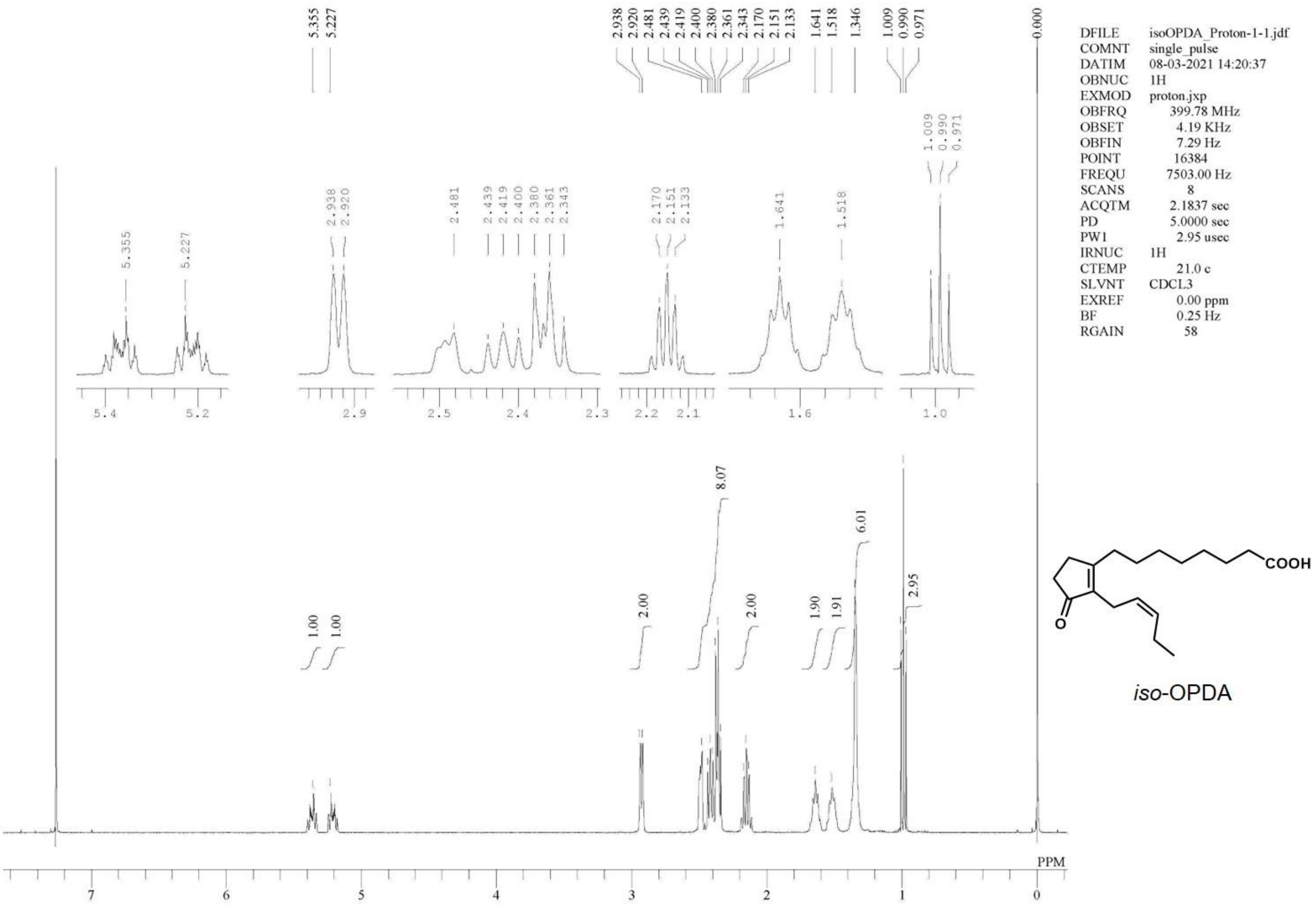

Supplementary Figure 1. ^{1}H NMR spectrum of *iso*-OPDA

^{1}H NMR spectrum was recorded on a JNM–ECS–400 spectrometer in deuterated chloroform using TMS as an internal standard.
