# Supplementary material for: Deciphering OPDA Signaling Components in the Momilactone-Producing Moss Calohypnum plumiforme
Source: Front Plant Sci. 2021 May 31;12:688565. doi: 10.3389/fpls.2021.688565 (PMC8201998; doi:10.3389/fpls.2021.688565)
Supplement: Supplementary Figure 2 — 13C NMR spectrum of iso-OPDA. [file Image_2.PDF]

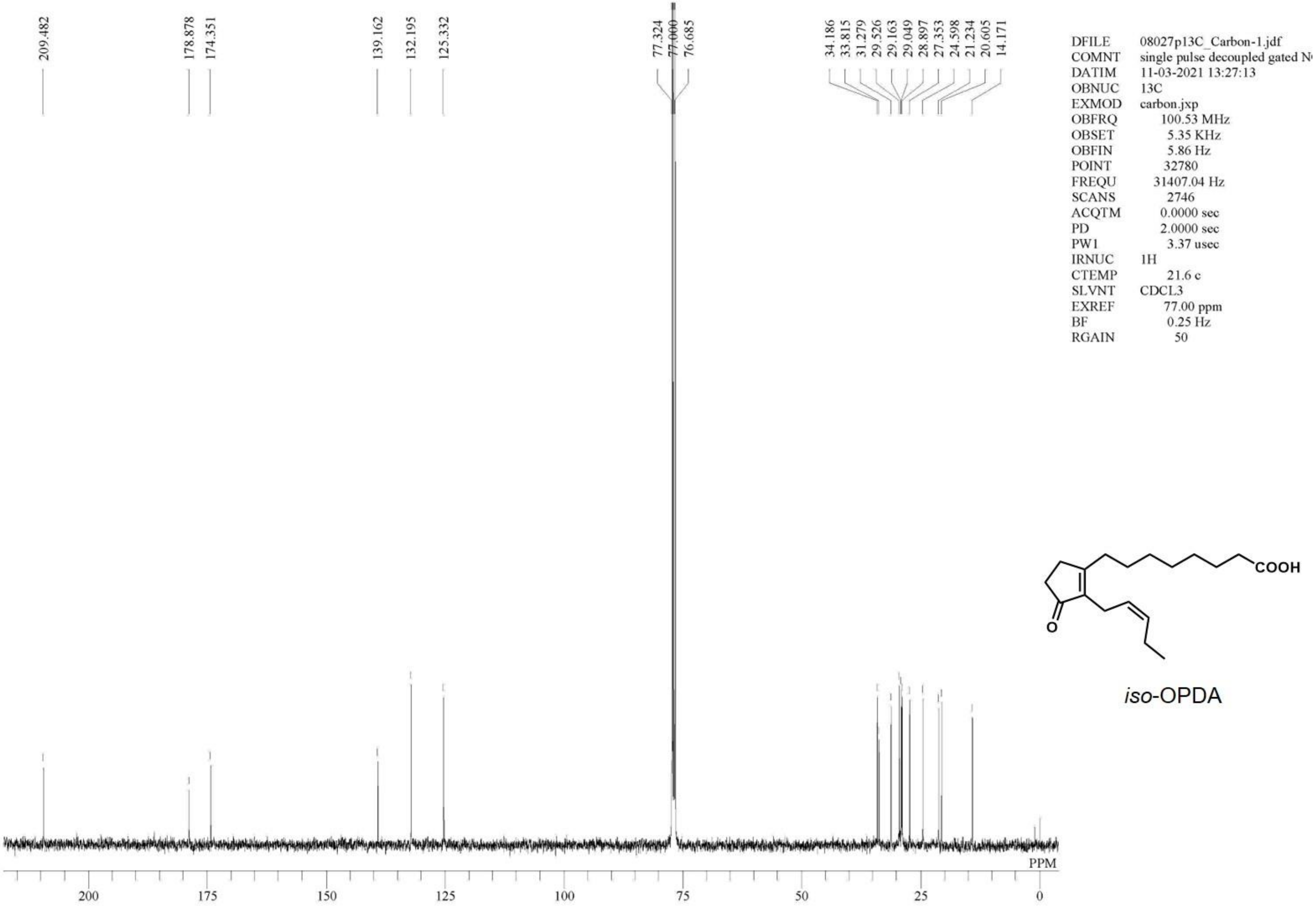

Supplementary Figure 2. ^{13}C NMR spectrum of *iso*-OPDA

^{13}C NMR spectrum was recorded on a JNM–ECS–400 spectrometer in deuterated chloroform using TMS as an internal standard.
