# Supplementary material for: Deciphering OPDA Signaling Components in the Momilactone-Producing Moss Calohypnum plumiforme
Source: Front Plant Sci. 2021 May 31;12:688565. doi: 10.3389/fpls.2021.688565 (PMC8201998; doi:10.3389/fpls.2021.688565)
Supplement: Supplementary Figure 3 — Sequence alignment of COIs. [file Image_3.PDF]

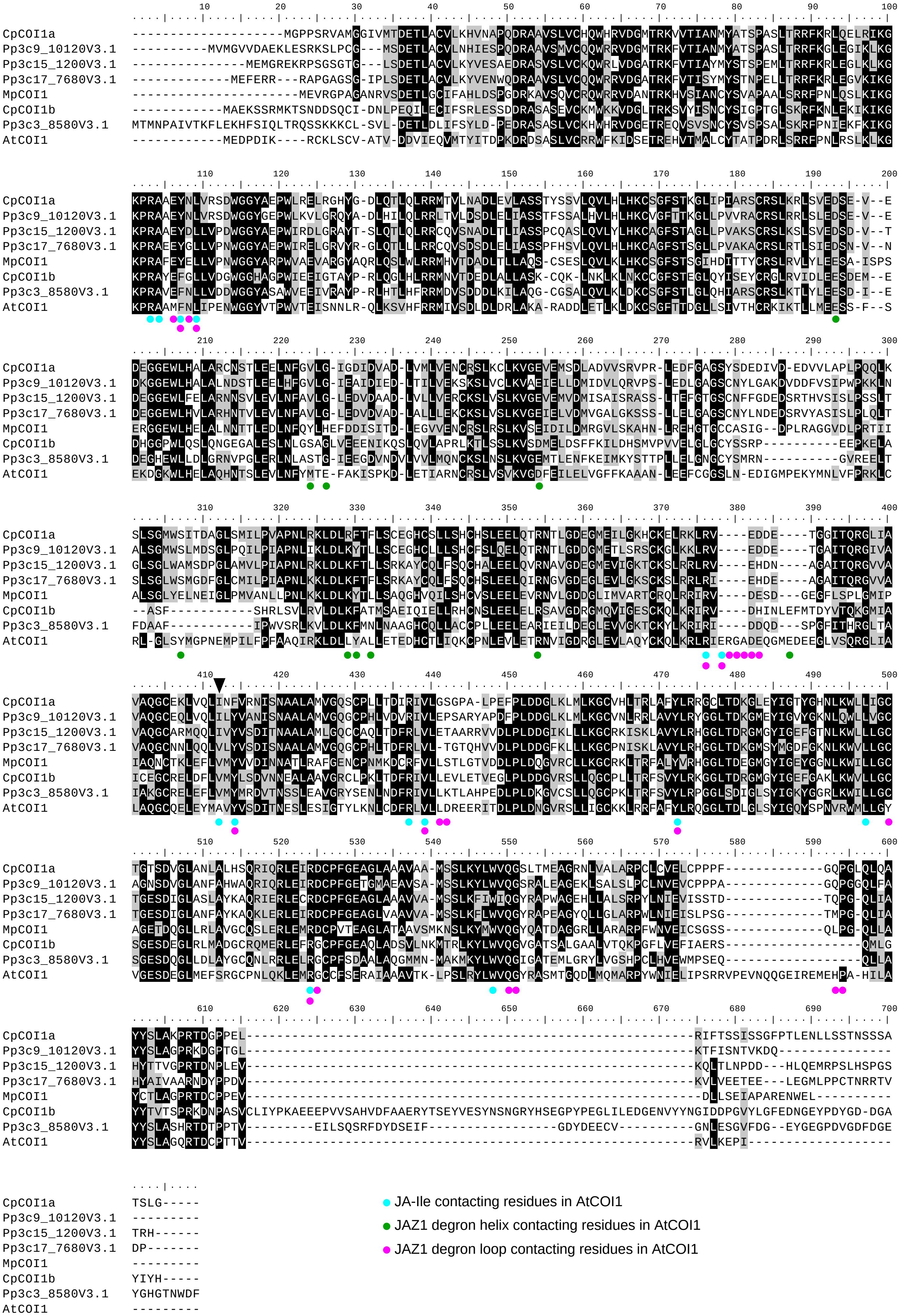

Supplementary Figure 3. Sequence alignment of COI1
Sequences of COI1 homologs were aligned by MEGA version X using Muscle. Sequence alignments were drawn using BioEdit software version 7.2.5. The Shading thresholds of identical or similar residues were 60%. Identical and similar residues were highlighted by black and gray, respectively. Ligand-, JAZ degron helix-, and JAZ degron loop- contacting residues in AtCOI1 were indicated by blue, green, and magenta dots, respectively. The residue essential for the recognition of dn-OPDA in MpCOI1 were indicated by a triangle.
