# Supplementary material for: Deciphering OPDA Signaling Components in the Momilactone-Producing Moss Calohypnum plumiforme
Source: Front Plant Sci. 2021 May 31;12:688565. doi: 10.3389/fpls.2021.688565 (PMC8201998; doi:10.3389/fpls.2021.688565)
Supplement: Supplementary Figure 4 — Differences between cloned CpMYC2s sequences and annotated sequences in the genome database. [file Image_4.PDF]

(A) 565-635 bp in *CpMYC2a* coding sequence

```
                            10        20        30        40        50        60        70
                    ....|....|....|....|....|....|....|....|....|....|....|....|....|....|....
MW775565            GCCCCCATCCATATCTGCACTCGAGCTCATTTGGCCAAGATGGCCGG---CATTCAGACAATTGTTTGTGTTCC
GWHTAMMO011051      GCCCCCATCCATATCTGCACTCGAGCTCATTTGGCCAAGGTTTCTTGTCCCTGCAGACAATTGTTTGTGTTCC
Clustal Consensus   ***************************************.*   *     *.* ********************
```

(B) 555-624 bp in *CpMYC2b* coding sequence containing 352 bp intron

```
                            10        20        30        40        50        60        70        80
                    ....|....|....|....|....|....|....|....|....|....|....|....|....|....|....|....|
MW775566            GCTAGAACAAGCCGACAAGGCCTCTAATCAAGTCTGCACACGAGCTAATTTAGCGAAG----------------------
GWHTAMMO007338      GCTAGAACAAGCCGACAAGGCCTCTAATCAAGTCTGCACACGAGCTAATTTAGCGAAG----------------------
Genome sequence     GCTAGAACAAGCCGACAAGGCCTCTAATCAAGTCTGCACACGAGCTAATTTAGCGAAGGTGAGATTCATGGATTTACTCT
Clustal Consensus   *********************************************************

                            90       100       110       120       130       140       150       160
                    ....|....|....|....|....|....|....|....|....|....|....|....|....|....|....|....|
MW775566            --------------------------------------------------------------------------------
GWHTAMMO007338      --------------------------------------------------------------------------------
Genome sequence     TTCAGACACTTGGCTTCAGATACGTAGTGCACGTAGTATCAACCTATATTTATTCAAATATATAGGACCATGTGCCGTCT
Clustal Consensus

                           170       180       190       200       210       220       230       240
                    ....|....|....|....|....|....|....|....|....|....|....|....|....|....|....|....|
MW775566            --------------------------------------------------------------------------------
GWHTAMMO007338      --------------------------------------------------------------------------------
Genome sequence     TGAAACATTACATTGTTGCTCTTTGAAGCCCGTGCTGAGTGAGTAGAGTAACGCATTCACATGTTTAGATTTTCTTTAGT
Clustal Consensus

                           250       260       270       280       290       300       310       320
                    ....|....|....|....|....|....|....|....|....|....|....|....|....|....|....|....|
MW775566            --------------------------------------------------------------------------------
GWHTAMMO007338      ------------------------------------------------------------------------GGTATGGG
Genome sequence     TTATGGTGGATTATGCAGATTATTTCTCTGATAGGAGTTTAAAGGATTGGAGATTGTGATTTTTGGTCGTAGGGTATGGG
Clustal Consensus
```

3′-End of annotated intron

```
                           330       340       350       360       370       380       390       400
                    ....|....|....|....|....|....|....|....|....|....|....|....|....|....|....|....|
MW775566            --------------------------------------------------------------------------------
GWHTAMMO007338      TGTACCGGGAACGAATACAGGTGAATGTTCTATAATACTCATGAAAATTTGTTTGTCGGTTGTGGTTGCATTTTGTCCTT
Genome sequence     TGTACCGGGAACGAATACAGGTGAATGTTCTATAATACTCATGAAAATTTGTTTGTCGGTTGTGGTTGCATTTTGTCCTT
Clustal Consensus
                                                         CTNAN
```

Putative branchpoint

```
                           410       420
                    ....|....|....|....|...
MW775566            -----------ATGGCCGGAATT
GWHTAMMO007338      CCCAAGAACAGATGGCCGGAATT
Genome sequence     CCCAAGAACAGATGGCCGGAATT
Clustal Consensus              ************
```

3′-End of actual intron

Supplementary Figure 4. Differences between cloned CpMYC2s sequences and annotated sequences in the genome database.
(A) Differences between cloned *CpMYC2a* and annotated sequences in the genome database. MW775565 is cloned sequence in this study. GWHTAMMO011051 is annotated transcript sequence.
(B) Differences between cloned *CpMYC2b* and annotated sequences in the genome database. MW775566 is cloned sequence in this study. GWHTAMMO007338 is annotated transcript sequence. Consensus sequences of 3′-end of intron (5′ -AG- 3′) are highlighted by black. Putative branchpoint is indicated by a box. Nucleotide sequences were aligned by CLUSTALW in GenomeNet (https://www.genome.jp/tools-bin/clustalw). Sequence alignments were drawn using BioEdit software version 7.2.5.respectively.
