# Supplementary material for: Deciphering OPDA Signaling Components in the Momilactone-Producing Moss Calohypnum plumiforme
Source: Front Plant Sci. 2021 May 31;12:688565. doi: 10.3389/fpls.2021.688565 (PMC8201998; doi:10.3389/fpls.2021.688565)
Supplement: Supplementary Figure 5 — Sequence alignment of MYC2. [file Image_5.PDF]

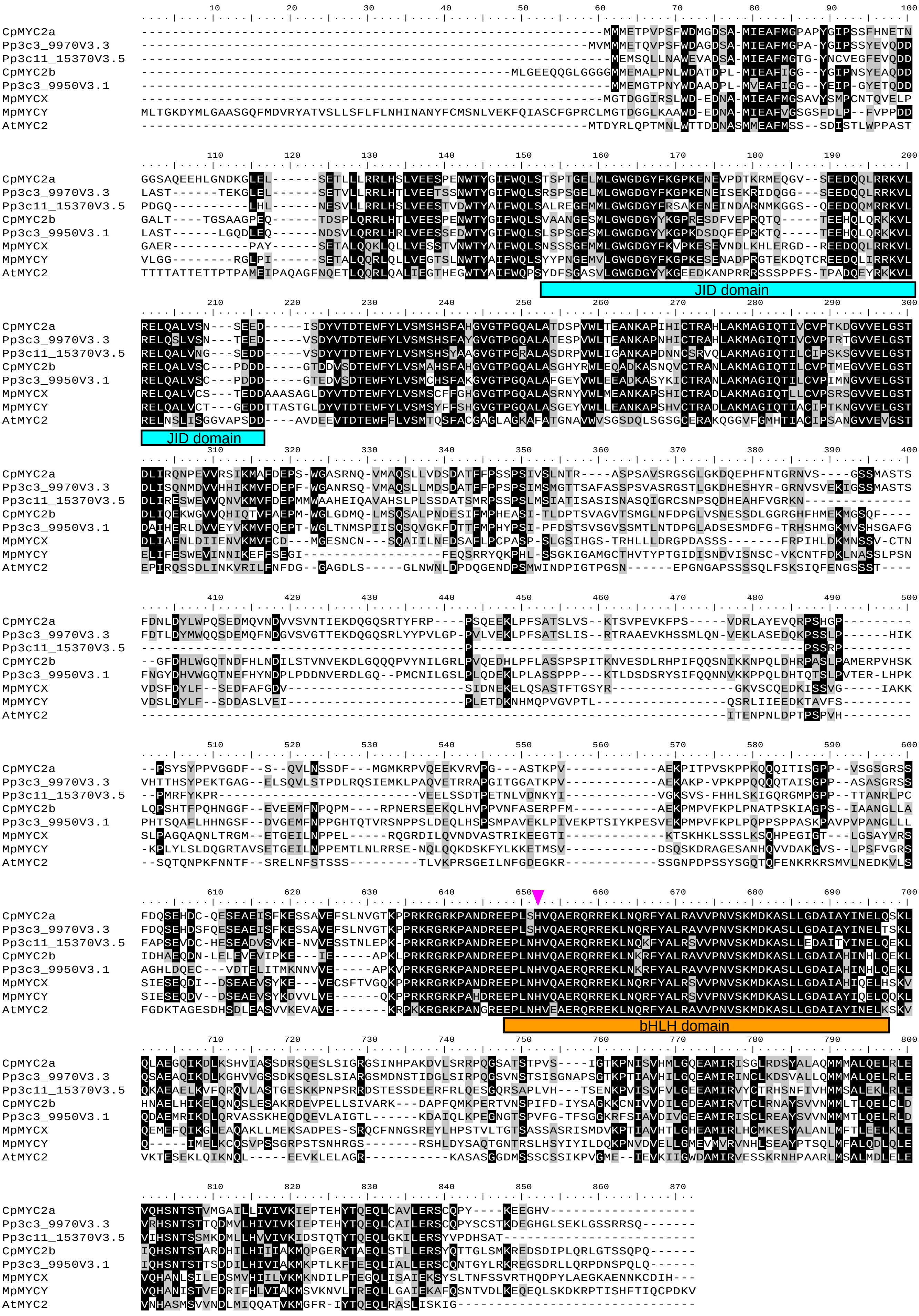

Supplementary Figure 5. Sequence alignment of MYC2

Sequences of MYC2 homologs were aligned by MEGA version X using Muscle. Sequence alignments were drawn using BioEdit software version 7.2.5. The Shading thresholds of identical or similar residues were 40%. Identical and similar residues were highlighted by black and gray, respectively. JID and bHLH domains were indicated by blue and orange squares, respectively. A magenta triangle indicates the histidine residue involving the binding to G-box (CACGTG)
