# Supplementary material for: Deciphering OPDA Signaling Components in the Momilactone-Producing Moss Calohypnum plumiforme
Source: Front Plant Sci. 2021 May 31;12:688565. doi: 10.3389/fpls.2021.688565 (PMC8201998; doi:10.3389/fpls.2021.688565)
Supplement: Supplementary Figure 6 — Expression of GST-CpMYC2s and FLAG-JAZs. [file Image_6.PDF]

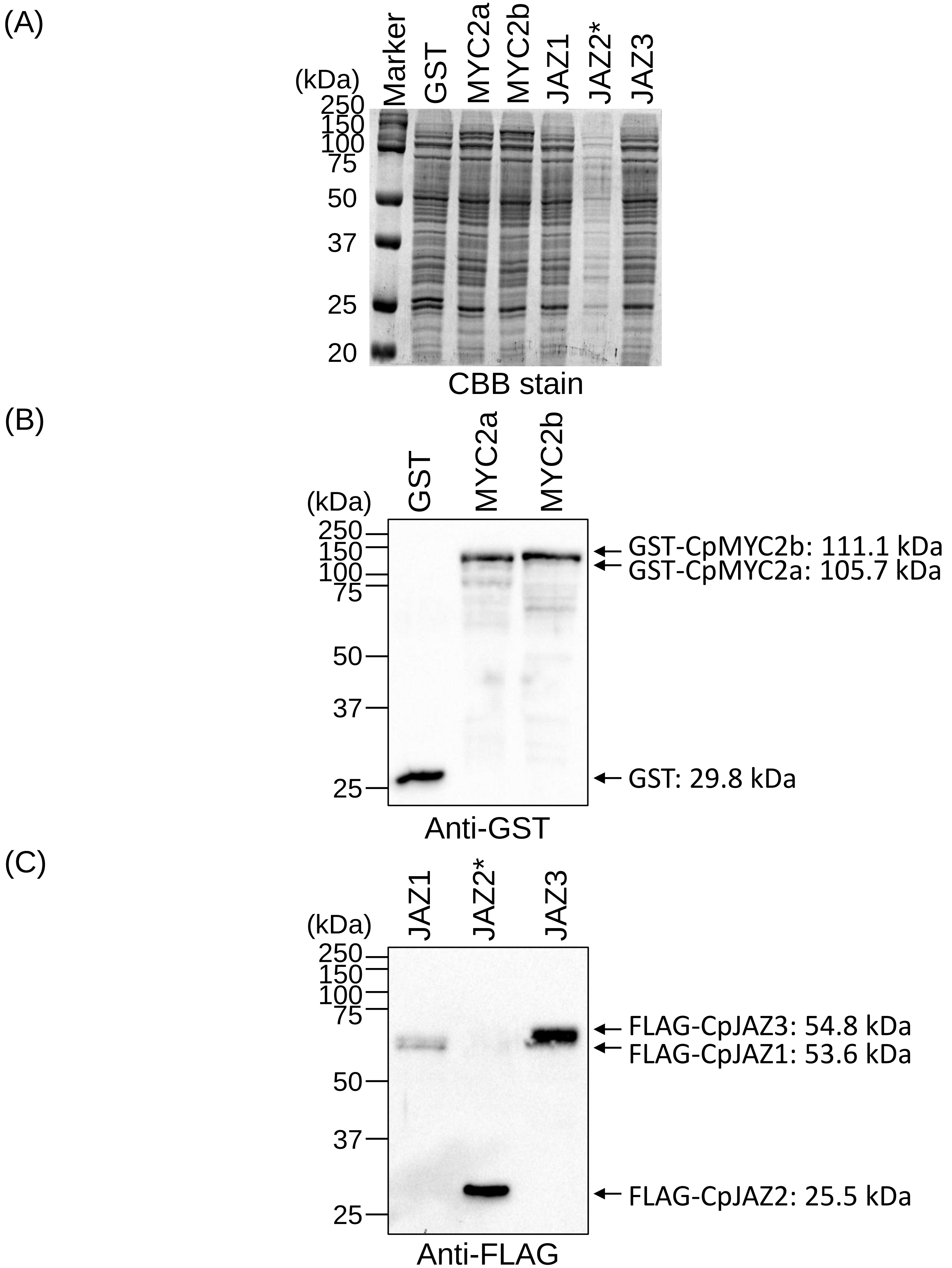

Supplementary Figure 6. Expression of GST-CpMYC2s and FLAG-JAZs.

(A) *In vitro* expressed proteins were subjected to SDS-PAGE and stained by CBB.

(B) *In vitro* expressed proteins were subjected to protein gel blot analyses and detected by anti-GST antibodies.

(C) *In vitro* expressed proteins were subjected to protein gel blot analyses and detected by anti-FLAG antibodies.

Marker: Precision Plus Protein Dual Color Standards (BioRad), *: the JAZ2 protein was diluted 5 times due to its high expression.
