# Supplementary material for: Deciphering OPDA Signaling Components in the Momilactone-Producing Moss Calohypnum plumiforme
Source: Front Plant Sci. 2021 May 31;12:688565. doi: 10.3389/fpls.2021.688565 (PMC8201998; doi:10.3389/fpls.2021.688565)
Supplement: Supplementary Table 1 — Primers used in this study. [file Table_1.PDF]

Supplementary Table 1: Primers used in this study

**Primers used in RACE**	
CpJAZ2 5'-RACE 1st PCR	5'-GATTACGCCAAGCTTTTGGGGAGATAGCCATGAAG-3'
CpJAZ2 5'-RACE 2nd PCR	5'-GATTACGCCAAGCTTGTCACTAGAGGAAGCCCCGCATGTC-3'
CpJAZ2 3'-RACE 1st PCR	5'-GATTACGCCAAGCTTCTTCATGGTCTACGCAAGCA-3'
CpJAZ2 3'-RACE 2nd PCR	5'-GATTACGCCAAGCTTGCCCCCTCTTACAGCACAACTCACC-3'

**Primers used in cloning of full length of cDNA**	
CpJAZ2 full length Forward	5'-GCAACCCACCTTCATCTCC-3'
CpJAZ2 full length Reverse	5'-ATATCAAAAGTCTAAAGCATTTTGC-3'

**Primers used in cloning of coding sequences**	
CpJAZ1 ORF Forward	5'-GACGATGACAAGGATATGGCGATGAGAGAGCCT-3'
CpJAZ1 ORF Reverse	5'-CGGGATCCTCGAGATTCACAACGAGGAGGATTCA-3'
CpJAZ2 ORF Forward	5'-GACGATGACAAGGATATGATGGCGGCTACGGGA-3'
CpJAZ2 ORF Reverse	5'-CGGGATCCTCGAGATCTAATTCCTTCTGGATGGAG-3'
CpJAZ3 ORF Forward	5'-GACGATGACAAGGATATGGCGAGGGAGCCTGTT-3'
CpJAZ3 ORF Reverse	5'-CGGGATCCTCGAGATCTATCGTGATGATGAATCGT-3'
CpCOI1a ORF Forward	5'-TTCCAGGGGCCCGATATGGGCCCCCCGAGCAGG-3'
CpCOI1a ORF Reverse	5'-CGGGATCCTCGAGATTCAGCCCAGAGAAGTGGC-3'
CpCOI1b ORF Forward	5'-TTCCAGGGGCCCGATATGGCGGAGAAGAGTTCG-3'
CpCOI1b ORF Reverse	5'-CGGGATCCTCGAGATCTAATGGTAGATGTAAGCTC-3'
CpMYC2a ORF Forward	5'-TTCCAGGGGCCCGATATGATGATGGAGACGCCAG -3'
CpMYC2a ORF Reverse	5'-CGGGATCCTCGAGATTTATACGTGACCCTCTTCC-3'
CpMYC2b ORF Forward	5'-TTCCAGGGGCCCGATATGTTAGGGGAGGAGCAG-3'
CpMYC2b ORF Reverse	5'-CGGGATCCTCGAGATTTATTGAGGTTGAGAGGAGG-3'

**Primers used in qRT-PCR**	
CpACT3 Forward	5'-CGAGCAGCATGAAGATCAAG-3'
CpACT3 Reverse	5'-GTACTCGCTCTTCGCAATCC-3'
CpJAZ1 Forward	5'-CTTCATGGTCTACGCAAGCA-3'
CpJAZ1 Reverse	5'-GGGCTGAATTGTCCGATAGA-3'
CpJAZ2 Forward	5'-TTGGGGAGATAGCCATGAAG-3'
CpJAZ2 Reverse	5'-TGCAACAAAAGCTGAATTGC-3'
CpJAZ3 Forward	5'-TCCACTTCAAGCAGGGAACT-3'
CpJAZ3 Reverse	5'-CAAATGGCACGTCATCGTAG-3'
